# Neurofascin stabilization by contactin-associated protein-like 2 and CTCF alleviates mitochondrial dysfunction in Schwann cells during facial nerve injury

**DOI:** 10.1016/j.jbc.2025.111090

**Published:** 2025-12-22

**Authors:** Jin Zhu, Xin Ouyang, Lifu Yu, YeMei Qian, JinYi Li, Xun Sheng, Yu Liu, FengFeng Jia

**Affiliations:** 1 Department of Oral and Maxillofacial Surgery, Affiliated Stomatology Hospital of Kunming Medical University, Kunming, Yunnan, China; 2 Stomatology Centre, The First People's Hospital of Yunnan Province, Kunming, Yunnan, China; 3 Department of Otolaryngology, The First Affiliated Hospital of Kunming Medical University, Kunming, Yunnan, China

**Keywords:** facial nerve injury, NFASC, Schwann cells, oxidative stress, I-125, CTCF

## Abstract

During the growth or treatment of oral and maxillofacial tumors, the facial nerve is susceptible to injury, resulting facial dysfunction. Iodine-125 (I-125) seed brachytherapy is widely used in the treatment of various tumors due to its precise and sustained radiation characteristics, yet it may also cause nerve damage. However, the underlying mechanism of I-125 seed implantation-induced facial nerve injury (FNI) remains unclear. Here, a rabbit FNI model was established, and the neural injury mechanism was further investigated through I-125 seed implantation combined with transcriptome sequencing. Results found that neurofascin (NFASC) expression was reduced in injured rabbit facial nerves, and the implantation of I-125 particles further decreased NFASC expression. Overexpression of NFASC repressed oxidative stress and mito-chondrial dysfunction in I-125-treated Schwann cells (SCs), promoted cell proliferation, migration, and expression of myelin genes. Mechanistic studies revealed a protein-protein interaction between contactin-associated protein-like 2 (CNTNAP2) and NFASC, with CNTNAP2 positively regulating NFASC levels. CCCTC-binding factor (CTCF), as a transcription factor, positively regulated CNTNAP2 transcription. Compared to the groups overexpressing either CTCF or CNTNAP2 alone, simultaneous overexpression of CTCF and CNTNAP2 demonstrated an enhanced inhibitory effect on the oxidative stress in I-125-treated SCs, promoted cell proliferation, migration, and expression of myelin genes. CTCF overexpression improved demyelination of the facial nerve in rabbits with I-125 seed implantation and transverse injury by promoting CNTNAP2 and NFASC. In conclusion, CTCF/CNTNAP2/NFASC represents a critical pathway through which SCs mediate facial nerve damage caused by I-125 seed implantation. This finding provides potential therapeutic targets for the repair of FNI.

Oral and maxillofacial tumors are common tumors of the head and neck, with malignant tumors being the most prevalent (1). During their growth, these tumors may compress or invade surrounding facial nerves (2). Tumor resection and radiotherapy are effective treatments for this disease, but these treatments may cause certain damage to the facial nerve. The facial nerve is primarily responsible for controlling sensation, movement, and facial expression muscles. It is a mixed nerve composed of motor, sensory, and parasympathetic fibers (3). Facial nerve damage can lead to facial paralysis, with common symptoms including facial muscle paralysis, incomplete eyelid closure, altered taste, and auditory abnormalities, which severely affect patients' daily life and work (4). However, due to the unique anatomical location of the facial nerve and the complexity of the regeneration process, promoting the repair of facial nerve damage is a challenging task.

Iodine (I)-125 brachytherapy involves the implantation of radioactive seeds into tumor tissue, subjecting the tumor to continuous low-energy γ-ray treatment (5). This therapy is characterized by a high local dose with a rapid dose fall-off at the edges, short irradiation time, and relatively few side effects. I-125 seed implantation has been used in the treatment of various tumors, including prostate cancer (6), cholangiocarcinoma (7), lung cancer (8), and salivary gland malignancies (9). Recent studies have indicated that I-125 seed implantation may damage surrounding tissues, muscles, and nerves (10). In previous research, we discovered that I-125 seeds could inhibit Schwann cells (SCs) proliferation and migration, exacerbating facial nerve injury (FNI) in rats (11). However, the pathological mechanism underlying facial nerve damage caused by I-125 seed implantation needs further clarification.

SCs are glial cells in the peripheral nervous system. Activated SCs participate in myelin formation, maintain the structure and function of nerve fibers, and regulate nerve conduction, playing a crucial role in the repair process of damaged nerves (12). A study has shown that decreased c-Jun expression and reduced secretion of inhibitory factors in senescent and chronically denervated SCs could impair axon regeneration after peripheral nerve injury (13). Promoting the proliferation, migration, and paracrine function of SCs could facilitate functional recovery from FNI in rats (14).

Transcriptome sequencing utilizes high-throughput sequencing technology to acquire sequence information for almost all transcripts of specific tissues and organs in a certain state, thereby aiding in the untangling of specific biological processes and the molecular mechanisms involved in the occurrence and development of diseases. Based on previous research, in this study, we employed transcriptome sequencing to screen for differentially expressed genes (DEGs) in rabbit facial nerve tissues with FNI and I-125 implantation. Through enrichment analysis, we identified neurofascin (NFASC), which was associated with myelin formation and axon guidance and whose expression was reduced in the I-125 implantation group. NFASC, a neural cell adhesion molecule, plays a crucial role in axon growth, fasciculation of nerve fibers, and the establishment and maintenance of the axon initial segment and Ranvier's nodes (15). It has been reported to be involved in the myelination process of SCs (16). Subsequently, we exposed SCs to I-125 seed radiation to investigate the effects of NFASC on SC function. Furthermore, we predicted potential regulatory factors of NFASC through databases, delving into the underlying regulatory mechanisms of I-125 seed implantation in FNI.

## Results

### The expression of NFASC decreased in rabbits with FNI implanted with I-125 seed

**Figure 1:**
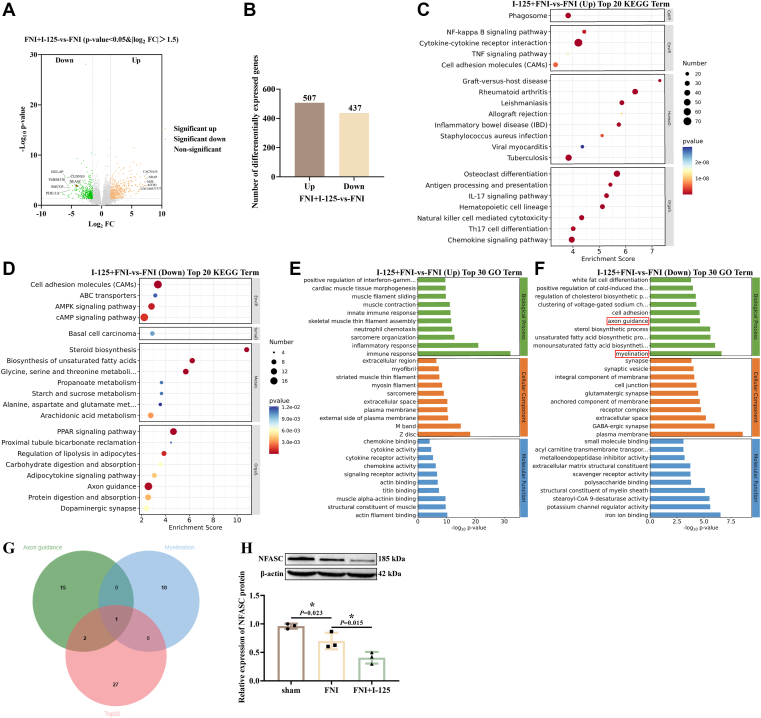
**The expression of NFASC decreased in rabbits with FNI implanted with I-125 seed.***A*, the heat map of DEGs. *B*, the quantification of DEGs. *C*, KEGG enrichment analysis of upregulated DEGs. *D*, KEGG enrichment analysis of downregulated DEGs. *E*, GO enrichment analysis of upregulated DEGs. *F*, GO enrichment analysis of downregulated DEGs. *G*, Venn analysis of myelin formation and axon guidance-related genes within the downregulated differential gene GO enrichment categories, along with the top 30 downregulated DEGs. *H*, detection of NFASC protein expression by Western blotting. ∗*p* < 0.05.

Transcriptome sequencing was performed on the facial nerves of rabbits in both the I-125 seed implantation group and the FNI group. DEGs were screened with the criteria of q-value < 0.05 and |log_2_ FC| ≥ 1.5, resulting in a volcano plot of differential genes as shown in Figure 1*A*. Compared to the FNI group, a total of 507 upregulated genes and 437 downregulated genes were identified in the I-125 group (Fig. 1*B*). KEGG enrichment analysis of the DEGs revealed that the upregulated DEGs were mainly enriched in items such as "cytokine-cytokine receptor interaction", "osteoclast differentiation", and "rheumatoid arthritis" (Fig. 1*C*), while the downregulated DEGs were primarily enriched in items including "steroid biosynthesis", "PPAR signaling pathway", and "cell adhesion molecules" (Fig. 1*D*). GO enrichment analysis indicated that the upregulated DEGs were enriched in GO items like "immune response", "Z disc", and "actin filament binding" (Fig. 1*E*), whereas the downregulated DEGs were enriched in terms such as "myelination", "plasma membrane", and "iron ion binding" (Fig. 1*F*).

Based on the aforementioned results, we conducted a Venn analysis on the DEGs related to myelination and axon guidance enriched in GO terms, and the top 30 downregulated genes. The intersection of these gene sets yielded NFASC (Fig. 1*G*). Western blotting was performed on rabbit facial nerve tissues, revealing that compared to the sham group, NFASC expression was reduced in the FNI group and further decreased in the I-125 seed implantation group relative to the FNI group (Fig. 1*H*). These findings suggest that the downregulation of NFASC in rabbits with FNI implanted with I-125 seeds is closely related to the progression of FNI.

### I-125 regulated SCs mitochondrial dysfunction and expression of myelin genes through NFASC

**Figure 2:**
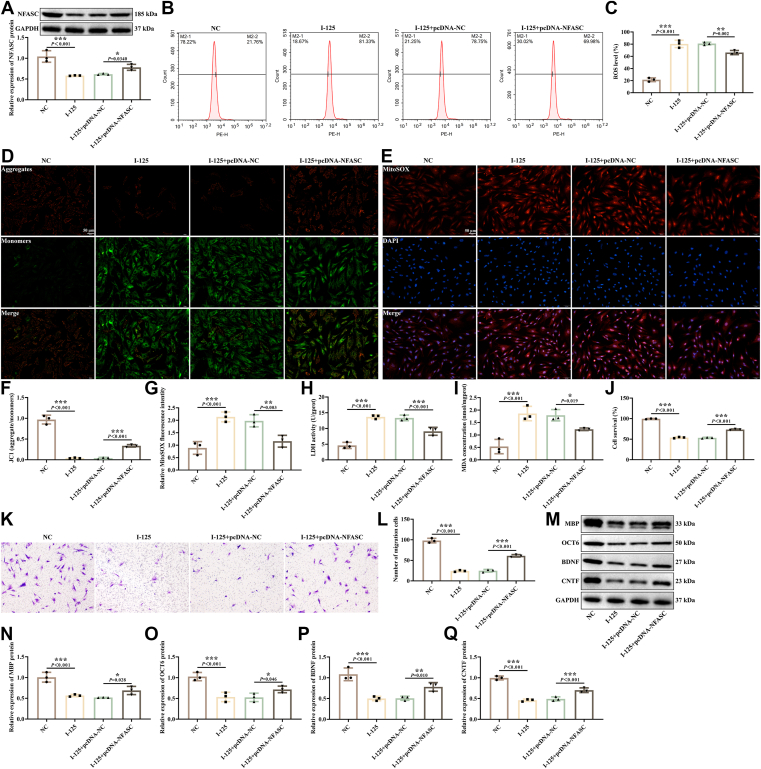
**I-125 regulated SCs mitochondrial dysfunction and expression of myelin genes through NFASC.***A*, detection of NFASC protein expression by Western blotting. *B* and *C*, detection of ROS in cells by flow cytometry. *D* and *F*, measurement of mitochondrial membrane potential in cells through JC-1 staining. *E* and *G*, detection of superoxide anion in cells *via* MitoSOX staining. *H* and *I*, measurement of LDH and MDA levels using kits. *J*, determination of cell proliferation activity using CCK-8. *K* and *L*, assessment of cell migration ability through the Transwell assay. *M–Q*, Western blotting analysis for the detection of MBP, OCT6, BDNF, and CNTF protein expression. ∗*p* < 0.05, ∗∗*p* < 0.01, ∗∗∗*p* < 0.001.

SCs play a pivotal role in nerve injury repair, as they are involved in the formation of myelin sheaths, maintain the structure and function of nerve fibers, and regulate neural conduction (17). In this study, we exposed human SCs to I-125 seeds radiation and transfected them with pcDNA-NC and pcDNA-NFASC to investigate the effect of NFASC expression on radiation-damaged SCs. Western blotting results found that the expression of NFASC was reduced in SCs treated with I-125 (Fig. 2*A*), while its expression was significantly higher in SCs transfected with pcDNA-NFASC compared to those transfected with pcDNA-NC. Flow cytometry results based on the fluorescent probe DCFH-DA indicated an increase in ROS levels in the I-125-treated SCs (Fig. 2, *B* and *C*), which was inhibited by the overexpression of NFASC. JC-1 staining revealed a decrease in MMP in the I-125 group (Fig. 2, *D* and *F*), which was increased by the overexpression of NFASC. MitoSOX staining and kits detection showed elevated levels of superoxide anion (Fig. 2, *E* and *G*), LDH (Fig. 2*H*), and MDA (Fig. 2*I*) concentrations in the I-125-treated SCs, effects that were mitigated by the overexpression of NFASC. Additionally, CCK-8, Transwell, and western blotting assays demonstrated that I-125 treatment inhibited cell proliferation (Fig. 2*J*), migration ability (Fig. 2, *K* and *L*), and MBP (Fig. 2, *M* and *N*), OCT6 (Fig. 2, *M* and *O*), BDNF (Fig. 2, *M* and *P*), and CNTF (Fig. 2, *M* and *Q*) proteins expression, while the overexpression of NFASC promoted SC proliferation, migration, and MBP, OCT6, BDNF, and CNTF expression. These data indicate that overexpression of NFASC can reduce oxidative stress induced by I-125 stimulation in SCs, promoting cell proliferation, migration, and expression of myelin genes.

### Protein–protein interaction (PPI) between NFASC and CNTNAP2

**Figure 3:**
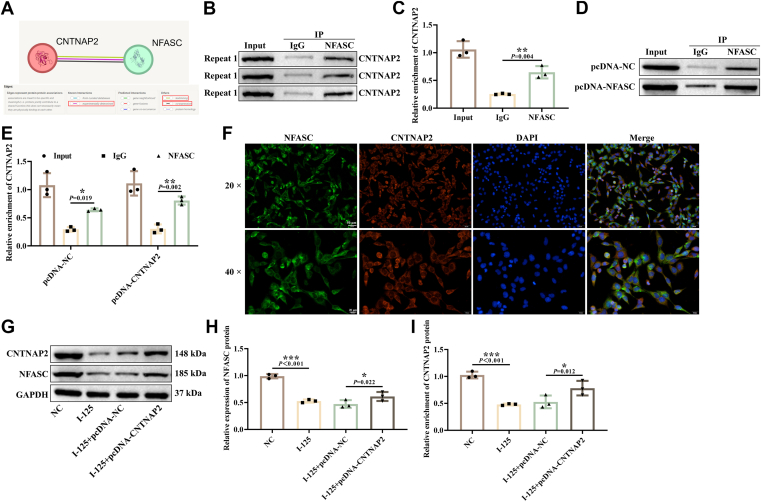
**PPI between NFASC and CNTNAP2.***A*, the STRING database predicted the protein-protein interaction between NFASC and CNTNAP2. *B* and *C*, Co-IP assay validating the binding of NFASC to CNTNAP2. *D* and *E*, Co-IP assay detecting the interaction between NFASC and CNTNAP2. *F*, IF experiment examining the localization of NFASC and CNTNAP2 in SCs. *G-I*, Western blotting analysis assessing the protein expression of NFASC and CNTNAP2. ∗*p* < 0.05, ∗∗*p* < 0.01, ∗∗∗*p* < 0.001.

Through predictions from the STRING database, a protein-protein interaction (PPI) between CNTNAP2 and NFASC was identified, with an interaction score of 0.722, indicating high confidence. This interaction is supported by strong evidence from textmining, experimentally determined data, and co-expression analyses (Fig. 3*A*). CNTNAP2 is involved in cell adhesion and recognition and plays an important role in the normal development and function of peripheral nerves (18). However, its role in SCs-mediated FNI has not yet been investigated. Further Co-IP detection indicated significant enrichment of CNTNAP2 with anti-NFASC antibodies (Fig. 3, *B*–*E*). IF staining demonstrated the colocalization of NFASC and CNTNAP2 on the cell membrane (Fig. 3*F*). Western blotting analysis revealed that the expression of CNTNAP2 and NFASC in the I-125 treatment group decreased (Fig. 3, *G*–*I*), overexpression of CNTNAP2 could upregulate the expression of both CNTNAP2 and NFASC. These findings reveal that CNTNAP2 positively regulates NFASC expression. Furthermore, existing studies have confirmed that CNTNAP2 is involved in the regulation of oxidative stress and inflammatory responses (19). Therefore, we propose the following hypothesis: CNTNAP2 may regulate oxidative stress and inflammatory responses in SCs through its PPI with NFASC and mediate the pathophysiological processes of FNI and repair in this context.

### CTCF regulated CNTNAP2 transcription

**Figure 4:**
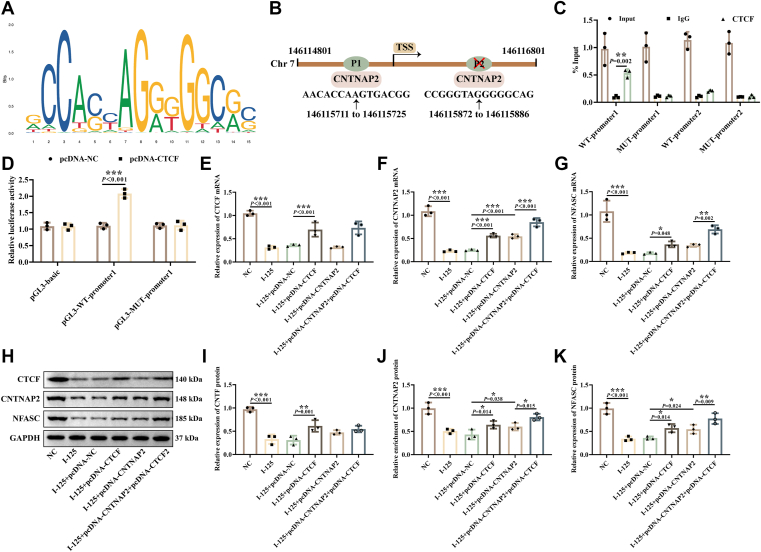
**CTCF regulated CNTNAP2 transcription.***A*, the CTCF motif predicted by the JASPAR database as potentially binding to CNTNAP2. *B*, the CNTNAP2 sequence predicted by the JASPAR database as binding to CTCF. *C*, CUT&RUN-PCR analysis to detect the binding region of CTCF to the CNTNAP2 promoter. *D*, dual-luciferase reporter assay to verify the binding of CTCF to CNTNAP2. *E–G*, mRNA levels of CTCF, CNTNAP2, and NFASC. *H–K*, protein levels of CTCF, CNTNAP2, and NFASC. ∗*p* < 0.05, ∗∗*p* < 0.01, ∗∗∗*p* < 0.001.

Next, we predicted by JASPAR database that CTCF may bind to the CNTNAP2 promoter as a transcription factor. The CTCF binding motif is shown in Figure 4*A*, and the CNTNAP2 promoter sequence is displayed in Figure 4*B*. The scores for promoter site 1 and site 2 were 6.855 and 6.743, respectively, with relative scores of 0.802 and 0.8001. CUT&RUN-PCR results indicated a significant enrichment of CTCF with CNTNAP2 promoter 1 (Fig. 4*C*). The dual-luciferase reporter gene assay further confirmed the targeting relationship between CTCF and CNTNAP2. Overexpression of CTCF enhanced the luciferase activity of wild-type CNTNAP2 (Fig. 4*D*), while it had no significant effect on the luciferase activity of mutant CNTNAP2. RT-qPCR (Fig. 4, *E*–*G*) and western blotting (Fig. 4, *H*–*K*) results revealed that CTCF, CNTNAP2, and NFASC levels in I-125-treated SCs were repressed; overexpression of CTCF promoted CTCF, CNTNAP2, and NFASC levels. Overexpression of CNTNAP2 promoted CNTNAP2 and NFASC expression. Meanwhile, the expression levels of CNTNAP2 and NFASC were elevated in the simultaneous overexpression of CTCF and CNTNAP2 group compared to the groups with overexpression of either CTCF or CNTNAP2 alone (Fig. 4, *E*–*K*). These findings suggest that CTCF functions as a transcriptional factor that positively regulates CNTNAP2 transcription. CNTNAP2, in turn, positively modulates NFASC expression *via* PPI.

### Overexpression of CTCF regulated I-125-induced SCs function *via* CNTNAP2/NFASC

**Figure 5:**
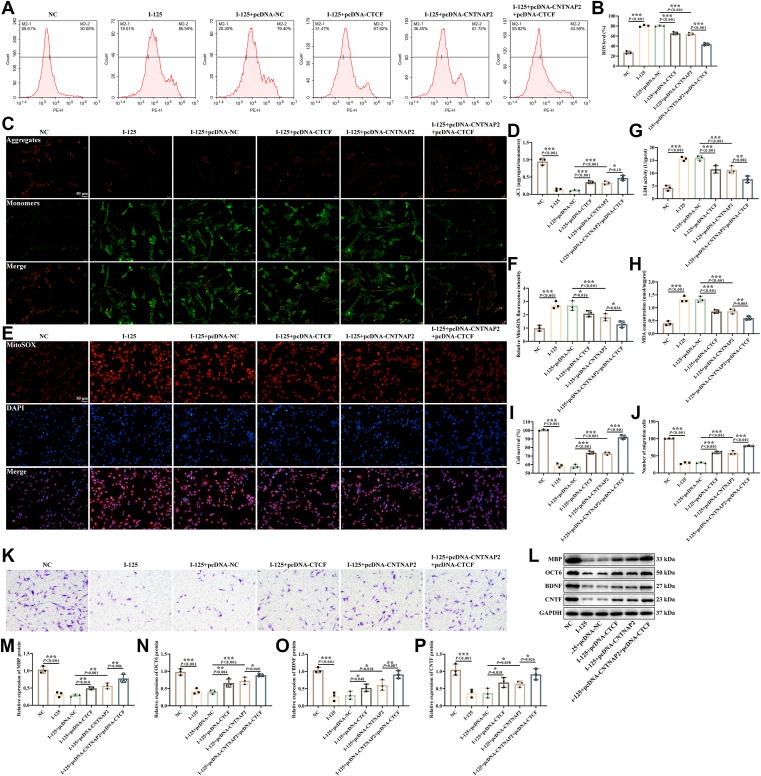
**Overexpression of CTCF regulated I-125-induced SCs function *via* CNTNAP2/NFASC.***A* and *B*, detection of ROS in SCs by flow cytometry. *C* and *D*, detection of mitochondrial membrane potential by JC-1 staining. *E* and *F*, detection of superoxide anion in cells by MitoSOX staining. *G* and *H*, detection of LDH and MDA using kits. *I*, cell proliferation activity. *J* and *K*, detection of cell migration by Transwell. *L–P*, detection of MBP, OCT6, BDNF, and CNTF proteins expression in cells by western blotting. ∗*p* < 0.05, ∗∗*p* < 0.01, ∗∗∗*p* < 0.001.

Subsequently, we investigated the role of CTCF in regulating the biological behavior of I-125-induced SCs through CNTNAP2/NFASC modulation. Compared to the overexpression of the NC group, the overexpression of CTCF or CNTNAP2 reduced ROS levels in I-125-treated SCs (Fig. 5, *A* and *B*), increased MMP (Fig. 5, *C* and *D*), and suppressed superoxide anion (Fig. 5, *E* and *F*), LDH (Fig. 5*G*), and MDA (Fig. 5*H*) levels in the cells. Simultaneous overexpression of CTCF and CNTNAP2 further potentiated the effects of overexpressing either CTCF or CNTNAP2 on SCs, inhibiting oxidative stress and mitochondrial dysfunction in the cells (Fig. 5, *A*–*H*).

The CCK-8 (Fig. 5*I*), Transwell (Fig. 5, *J* and *K*), and Western blotting (Fig. 5, *L*–*P*) experiments demonstrated that overexpression of CTCF or CNTNAP2 promoted proliferation, migration, neurotrophin production, and expression of myelin genes in I-125-treated SCs. Additionally, the co-overexpression of CTCF and CNTNAP2 resulted in higher levels of cell proliferation, migration, neurotrophin production, and expression of proteins related to myelin formation compared to groups that overexpressed only CTCF or CNTNAP2 (Fig. 5, *I*–*P*). These data indicate that overexpression of CTCF enhances the expression of CNTNAP2 and NFASC, thereby suppressing oxidative stress and mitochondrial dysfunction in I-125-treated SCs, ultimately facilitating cell proliferation, migration, and expression of myelin genes.

### The regulatory effect of overexpressed CTCF on FNI *in vivo*

**Figure 6:**
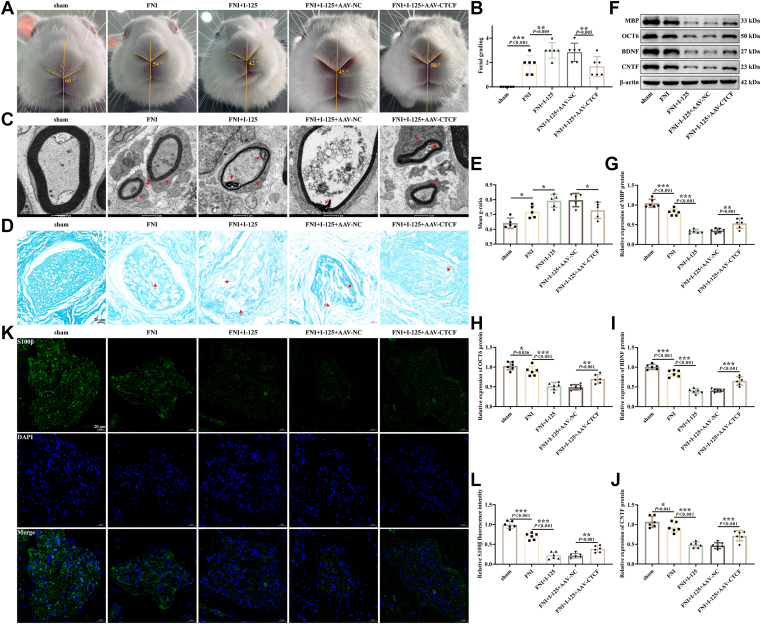
**The regulatory effect of overexpressed CTCF on FNI *in vivo*.***A*, representative images of a rabbit's face. *B*, Facial function score. *C*, representative TEM images of rabbit facial nerve. *Red arrow*: indicates the perimyelin sheath and rupture. *D*, representative LFB staining images of rabbit facial nerve. *Red arrow*: indicates the perimyelin sheath and rupture. *E*: *F–J*, detection of MBP, OCT6, BDNF, and CNTF proteins expression by Western blotting. *K* and *L*, the fluorescence images of S100β detected by IF staining. ∗*p* < 0.05, ∗∗*p* < 0.01, ∗∗∗*p* < 0.001.

To investigate the role of CTCF in facial nerve transection injury and I-125 implant injury, we constructed an FNI rabbit model by implanting I-125 seeds around the injured nerve in rabbits, and simultaneously injecting adeno-associated virus stably overexpressing CTCF into the rabbits' faces. Observation of the rabbits' faces revealed that compared to the sham group, the rabbits in the FNI group exhibited delayed eye movement and eyelid closure, weakened whisker movement (Fig. 6*A*), and an increase in facial function scores on the affected side (Fig. 6*B*). In the I-125 group, the delay in eye movement and eyelid closure on the affected side of the rabbits was more pronounced than in the FNI group, leading to an increase in facial function scores. In the overexpression of the CTCF group, the delay in the corneal reflex was reduced compared to the overexpression of the NC group, the deviation of the nose tip was decreased, and facial function scores were lowered.

TEM (Fig. 6*C*) and LFB (Fig. 6, *D* and *E*) staining revealed irregularly arranged, shrunken, and partially ruptured myelin sheaths in the FNI group, along with an increased g-ratio. These pathological features—demyelination, disrupted continuity, and more severe myelin malformation—were exacerbated in the I-125 implantation group, which exhibited a further elevation in the g-ratio. By contrast, CTCF overexpression improved the myelin structure, resulting in relatively intact sheaths, reduced demyelination, and a lower g-ratio. Western blotting analysis indicated decreased expression of MBP, OCT6, BDNF, and CNTF proteins in the FNI group (Fig. 6, *F*–*J*), with even lower expression in the I-125 group. Overexpression of CTCF was found to increase the expression of MBP, OCT6, BDNF, and CNTF. IF staining was used to detect the SCs marker S100β. Fluorescence intensity of S100β was lower in the FNI group compared to the sham group. Implantation of I-125 seeds further suppressed S100β fluorescence, while overexpression of CTCF enhanced it (Fig. 6, *K* and *L*).

**Figure 7:**
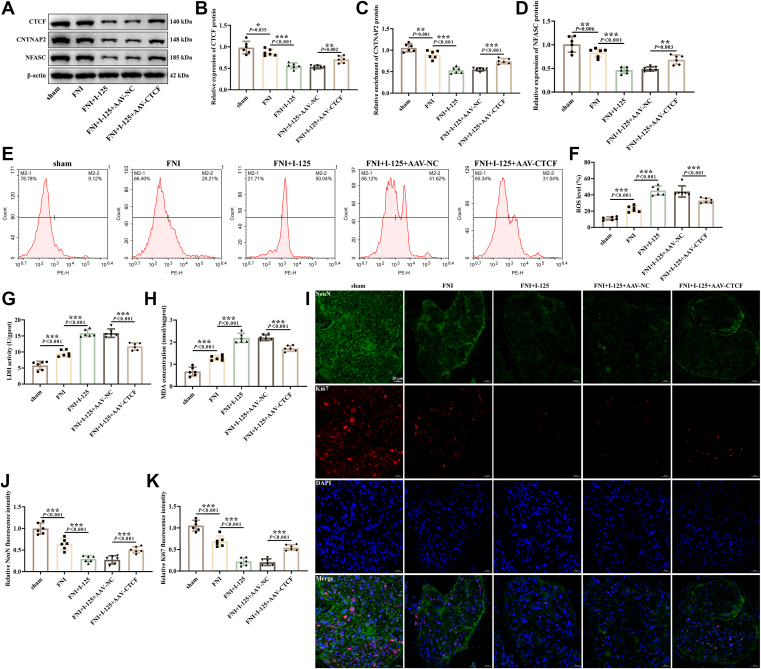
**Regulatory Effect of CTCF overexpression on oxidative stress and neurons in rabbits with FNI.***A–D*, detection of CTCF, CNTNAP2, and NFASC proteins expression by western blotting. *E* and *F*, detection of ROS in facial nerve tissue by flow cytometry. *G* and *H*, detection of LDH and MDA in the tissue using kits. *I–K*, IF staining was conducted to identify NeuN and Ki67 fluorescence intensity in the tissue. ∗*p* < 0.05, ∗∗*p* < 0.01, ∗∗∗*p* < 0.001.

Additionally, the expression of CTCF, CNTNAP2, and NFASC was reduced in the FNI rabbits (Fig. 7, *A*–*D*), and I-125 implantation further decreased the expression of CTCF, CNTNAP2, and NFASC, overexpression of CTCF upregulated their expression. The results of flow cytometry and kits indicated an increase in ROS (Fig. 7, *E* and *F*), LDH (Fig. 7*G*), and MDA (Fig. 7*H*) in the FNI group. I-125 seed implantation promoted the levels of ROS, LDH, and MDA. However, overexpression of CTCF suppressed their levels (Fig. 7, *E*–*H*). Through IF staining of Ki67 and neuronal markers NeuN, it was found that NeuN and Ki67 were reduced in the FNI group (Fig. 7, *I*–*K*), further decreased in the I-125 group, and overexpression of CTCF could promote the expression of NeuN and Ki67. These data suggest that overexpression of CTCF exerts a protective effect on rabbit facial nerves damaged by transection and I-125 implantation through regulating CNTNAP2/NFASC.

## Discussion

FNI is a common occurrence in oral and maxillofacial trauma, often resulting from the growth and treatment of oral and maxillofacial tumors (20, 21). The therapeutic approaches for oral and maxillofacial tumors encompass surgical intervention, radiotherapy, chemotherapy, targeted and immunotherapy, as well as combined modalities. The central strategy of combined therapy, which involves the integration of surgery and radiotherapy, elevates both local tumor control rates and patient survival rates. Conventional radiotherapy can harm healthy tissues, leading to side effects such as oral mucositis, radiation dermatitis, and osteonecrosis. Brachytherapy can reduce the occurrence of postoperative adverse reactions in patients (22). Radioactive I-125 seed implantation locally controls tumor growth by inducing DNA strand breaks in cancer cells. However, the implantation of I-125 seeds can also damage surrounding normal nerve tissues. If the radiation released by the seeds reaches peripheral nerves, it may trigger radioactive inflammatory responses and fibrosis. Additionally, long-term radiation exposure can exacerbate nerve damage. In this study, we performed transcriptome sequencing on facial nerve tissue, combined with bioinformatics analysis and experimental validation, to investigate the molecular mechanisms by which I-125 seeds exacerbate FNI through their effects on SCs oxidative stress and mitochondrial dysfunction.

After FNI, Wallerian degeneration occurs, and the injury signal induces SCs to transform into repair SCs. The functions of these repair cells include recruiting macrophages, clearing cell debris, forming Büngners bands, and guiding axon regeneration and extension (23). Following axon regeneration, the repair SCs revert to mature SCs, enabling the regeneration of axonal myelin sheaths and restoring effective nerve conduction necessary for sensory and motor functions (24). Therefore, promoting the proliferation and migration of SCs is significant for nerve injury repair. In this study, through transcriptome sequencing and bioinformatics analysis, we discovered that NFASC was associated with myelin formation and axon guidance. NFASC expression was inhibited in the facial nerve of rabbits with FNI and even lower in rabbits with transverse nerve injury implanted with I-125 seed. Overexpression of NFASC could alleviate oxidative stress and mitochondrial dysfunction in SCs treated with I-125, promoting cell proliferation, migration, neurotrophin production, and expression of myelin genes.

NFASC is a member of the immunoglobulin superfamily and regulates cell adhesion, migration, axonal growth, and myelin formation, which are closely related to the development and function of the nervous system. Ablation of NFASC leads to the loss of axon-glia connections, resulting in the failure to separate axonal domains, a decrease in nerve conduction velocity, demyelination of axons, and ultimately axonal degeneration (25). A recent study has demonstrated that NFASC exhibits high mobility after being recruited to the axonal membrane, diffusing bidirectionally until it becomes anchored at the axon initial segment through interaction with AnkG (26). The absence of NFASC can lead to the dissociation of the axon initial segment in mice (27). In this study, we observed a decrease in NFASC expression in both SCs treated with I-125 seeds and in the facial nerve implanted with I-125 seeds. Overexpression of NFASC was found to promote the proliferation and migration of SCs. Additionally, there was a PPI between NFASC and CNTNAP2, and the overexpression of CNTNAP2 enhanced the expression of NFASC.

CNTNAP2 is a single-pass transmembrane protein that forms a protein network at synapses and axo-glial contacts by binding to protein partners. The ability of CNTNAP2 to recruit partners is related to its spatial position within cells. In myelinated axons, the extracellular domain of CNTNAP2 binds to the adhesion molecule Contactin 2 (CNTN2), forming a molecular bridge that spans the extracellular space, while the cytoplasmic tail of CNTNAP2 recruits K^+^ channels, mediating neurotransmission (28, 29). At synapses, CNTNAP2 on the presynaptic membrane binds to CNTN2 on the postsynaptic membrane, forming a trans-synaptic bridge that spans the synaptic cleft (30). CNTNAP2 is crucial for maintaining normal network activity and synaptic transmission. Its absence leads to a reduction in dendritic branching, as well as a decrease in the number of inhibitory interneurons, excitatory synapses, and inhibitory synapses (31). Myelinating SCs and oligodendrocytes segment axons into sections separated by Ranvier nodes, and the absence of CNTNAP2 results in widened nodes of Ranvier (32), leading to delayed or blocked neural conduction. In this study, we discovered that CNTNAP2 expression was reduced in I-125-treated SCs and in injured facial nerves, positively regulating the expression of NFASC. Overexpression of CNTNAP2 repressed I-125-induced oxidative stress and mitochondrial dysfunction in SCs, promoted SCs proliferation, migration, and expression of myelin genes. Furthermore, through database analysis and experimental confirmation, we found that CNTNAP2 was transcriptionally regulated by CTCF, and overexpression of CTCF enhanced CNTNAP2 expression.

CTCF is a highly conserved zinc finger protein that plays a crucial role in chromatin structure and transcriptional regulation. It is essential for neuronal development, and its absence leads to defects in dendritic branching and dendritic spine density in mice (33). Wang *et al.* (34) discovered that CTCF was upregulated during the differentiation of SCs, restricting chromatin accessibility at gene loci associated with the immature SCs program. Simultaneously, by recruiting the PRC2/SUZ12 inhibitory complex, CTCF silences differentiation inhibitory signals, thereby promoting the expression of myelination genes. The absence of CTCF leads to increased chromatin accessibility and disrupts SCs differentiation and myelin formation. The deletion of CTCF in sciatic nerve or dorsal root ganglion neurons impairs neuronal regeneration (35), while CTCF and YY1 synergistically promote axon regeneration in damaged neurons (36). The results of this study indicate that CTCF expression is downregulated both in facial nerve tissues injured by I-125 seed implantation and in I-125-treated SCs. In the cellular model, overexpression of CTCF effectively suppressed I-125-induced oxidative stress and mitochondrial dysfunction. *In vivo*, CTCF overexpression also alleviated FNI caused by I-125 implantation and transection. Histological analysis revealed a reduction in the number of S100β-positive SCs and NeuN-labeled neurons following FNI, whereas CTCF overexpression significantly increased the fluorescence intensity of both markers. Combined with cellular experimental data, it is suggested that CTCF overexpression may improve the functional state of SCs, thereby providing a favorable microenvironment for neuronal survival and regeneration.

There exists a well-established bidirectional and direct association between oxidative stress and inflammatory responses, which can form a mutually reinforcing positive feedback loop (37). On one hand, oxidative stress can directly activate key pro-inflammatory signaling pathways such as NF-κB, thereby initiating and amplifying inflammatory responses. On the other hand, activated inflammatory cells also produce large amounts of reactive oxygen species, further exacerbating the level of oxidative stress. Previous studies have indicated that CTCF is involved in the regulation of inflammatory responses in systems such as the liver and neurons (38, 39). This study demonstrates that CTCF overexpression significantly alleviates oxidative stress in FNI induced by I-125 particles. Based on the aforementioned mechanistic relationship, it is reasonably hypothesized that CTCF may also indirectly regulate the inflammatory microenvironment following FNI by suppressing oxidative stress. Further investigations will be conducted to thoroughly validate this hypothesis.

In summary, our study indicates that I-125 seed implantation may inhibit the proliferation, migration, and myelin formation of SCs by inducing oxidative stress. This process likely occurs through the modulation of CTCF/CNTNAP2/NFASC. The overexpression of CTCF promotes CNTNAP2 transcription and NFASC expression, thereby exerting a protective effect on damaged facial nerves.

## Limitations

Despite its strengths, our research is not without limitations. In the experimental design of this study, due to the lack of established research foundations for constructing cellular models of FNI at the time, the cellular experiments focused solely on the radiation injury process of SCs induced by I-125, without establishing a cellular model simulating FNI. Subsequent studies will draw upon recent literature reports (*e.g.*, the LPS-induced method) to further explore and optimize the construction strategy of such models, aiming to provide a more robust experimental basis for related research.

During the establishment of the FNI cell model using I-125 particle irradiation, four I-125 seeds were placed in each well of a 6-well plate. Although the plate was regularly rotated to promote uniform radiation distribution, it remained difficult to ensure homogeneity of the radiation exposure dose across all wells. In subsequent studies, we will further optimize the irradiation conditions to improve the consistency of the radiation dose and enhance the reliability of the experimental results.

Although the present study has preliminarily validated the relevant conclusions using the available data, it must be acknowledged that the sample size per group in the current cellular and animal experiments remains relatively limited. Increasing the sample size would help enhance the statistical power, thereby allowing for a more reliable confirmation of the magnitude of the observed effects and further improving the robustness of the research findings. In subsequent work, we will expand the sample size to conduct more in-depth validation.

Moreover, this study only preliminarily investigated the effects of the CTCF/CNTNAP2/NFASC axis on SCs function through overexpression methods, and has not yet utilized knock-out experiments to verify the functional necessity of this pathway. Its specific mechanism of action still requires further elucidation. Meanwhile, whether NFASC acts as a necessary or sufficient condition in the CTCF-mediated response, and whether its mode of action is direct or indirect, also requires further clarification. It is worth noting that, given CTCF’s broad roles in chromatin structure and gene expression regulation, its overexpression may lead to pleiotropic and off-target effects. Therefore, systematic and rigorous evaluation of its safety and specificity will be essential in future clinical translation studies.

## Experimental procedures

### Experimental animals

Thirty-nine male Japanese white rabbits (2–2.5 kg, 3–4 months) were provided by the Experimental Animal Department of Kunming Medical University (SCXK (Dian) 2020–0004). All animal experiments complied with ARRIVE (Animal Research: Reporting of In Vivo Experiments) guidelines and were approved by the Experimental Animal Ethics Committee of Kunming Medical University (kmmu20230921). The rabbits were housed in an environment maintained at 18 to 22 °C with 50%-70% humidity, where they had free access to food and water. The animals were acclimated for 1 week before the commencement of the experiments.

### Construction of the FNI model and I-125 seed implantation

The animal experiment was divided into two parts: Part 1: sham, FNI, and FNI+I-125 group, n = 3; the sample was used for transcriptome sequencing. Part 2: sham group, FNI group, FNI+I-125 group, FNI+I-125+adeno-associated virus (AAV)-NC (Shanghai Genomeditech) group, and FNI+I-125+AAV-CCCTC-binding factor (CTCF, Shanghai Genomeditech, GPAAV-CMV-Rabbit_CTCF-T2A-eGFP-WPRE, 1.07 × 10^12^ VG/ml) group, with n = 6 for each group.

Rabbits were anesthetized with 3% pentobarbital sodium (1 ml/kg) injected through the marginal ear vein. With the left side of the rabbit's face facing up and fixed to the operating table, the surgical area was shaved using a depilator and sterilized with iodophor. An arcuate incision was made behind the ear, subcutaneous tissue and muscle were separated, and the main trunk of the facial nerve behind the stylomastoid foramen was exposed. The facial nerve was cut with scissors, and the subcutaneous tissue and skin were sutured and routinely disinfected. In the FNI+I-125 group, an I-125 seed (Shanghai Xinke, 0.8 mCi, half-life of approximately 60.1 days) was implanted 0.3 to 0.5 cm away from the facial nerve during skin suturing. In the sham group, the skin was sutured after exposing the main trunk of the facial nerve. For the FNI+I-125+AAV-NC and FNI+I-125+AAV-CTCF groups, AAV vectors and overexpressed CTCF AAV were injected subcutaneously around the rabbit's facial nerve 14 days before FNI modeling, with each rabbit receiving 1.07 × 10^12^ vg/ml of 50 μl. Animals were routinely fed after surgery, and facial function scores were assessed 14 days later. Rabbits were euthanized with an overdose of anesthetic, and their facial nerves were harvested for experimentation.

### Library construction and RNA sequencing

Total RNA was extracted from the facial nerve using TRIzol reagent (Thermo Fisher Scientific, 15596018CN), followed by digestion of DNA with deoxyribonuclease. Eukaryotic mRNA was enriched using magnetic beads coated with Oligo dT, and fragmented into short segments by adding lysis buffer. cDNA was synthesized using these mRNA fragments as templates and purified with a kit. These purified double-stranded cDNAs underwent end repair, addition of an A-tail, ligation of sequencing adapters, and PCR amplification using primers on the adapters. The constructed library was tested using an Agilent 2100 Bioanalyzer and sequenced using an Illumina HiSeq X 10 sequencer.

In this study, reference-based transcriptome sequencing was performed on six samples, generating a total of 40.87 Gb of high-quality clean data. The amount of valid data per sample ranged from 6.54 Gb to 6.96 Gb. The percentage of bases with a quality score of Q30 or higher exceeded 93.73% in all samples, reaching a maximum of 93.92%, and the average GC content was 56.67%. By aligning the sequencing reads to the reference genome, genome mapping rates between 85.99% and 87.42% were obtained across the samples. Analysis of gene body coverage evenness indicated that the reads were distributed relatively uniformly along the full length of the gene transcripts, with no significant 5′ or 3′ bias observed.

### Analysis of sequencing data

The original image data files obtained from sequencing were converted into the original sequencing sequences' raw data by base recognition analysis, and stored in FASTQ file format. Sequencing data was obtained through sequence structure analysis, library quality assessment, and comparison with the reference genome. The number of mapped reads and transcriptome length in the samples were normalized. Fragments per kilobase of transcript per million mapped reads (FPKM) was used as an indicator to measure gene expression levels. Statistical significance was determined by a |log FC (fold change) | > 1.5 and a q-value < 0.05.

### GO and KEGG enrichment analysis

DEGs were analyzed using Gene Ontology (GO) and the Kyoto Encyclopedia of Genes and Genomes (KEGG) databases. The significance of DEGs enrichment in each GO and KEGG term was calculated using the hypergeometric algorithm. DEGs were identified based on a threshold of |log FC| > 1.5 and *p* < 0.05.

### Total protein extraction and western blotting experiments

Tissues and cells were lysed using RIPA buffer (Shanghai Beyotime, P0013 B) containing phosphatase inhibitors and protease inhibitors. The protein sample concentrations were measured using an ultra-micro spectrophotometer. The 10% Sodium dodecyl sulfate (VETEC, V900859) - polyacrylamide gel electrophoresis was performed to separate the proteins (80 μg), which were then transferred to PVDF membranes (Sigma-Aldrich, IPVH00010, MO, USA). After blocking with 5% skim milk for 2 h, the PVDF membranes were incubated with primary antibodies: mouse anti-NFASC (NeuroMab, 75–027, CA, USA), rabbit anti-MBP (Beijing Bioss, bs-24827R), rabbit anti-BDNF (Beijing Bioss, bs-4989R), rabbit anti-OCT6 (Wuhan Proteintech, 18997-1-AP), rabbit anti-CNTF (Shanghai Abcam, ab190985), mouse anti-β-actin (Beijing ZHGB-Bio, TA-09), mouse anti-GAPDH (Beijing ZHGB-Bio, TA-08), rabbit anti-CTCF (Shanghai Abcam, ab128873), rabbit anti-contactin-associated protein-like 2 (CNTNAP2, Shanghai Abcam, ab153856). The rinsed PVDF membranes was incubated with second antibodies (HRP-conjugated goat anti-rabbit IgG (H&L) Wuhan Servicebio, GB23303; Goat Anti-Mouse IgG HRP, Shanghai Abmart, M21001S) at 25 °C for 2 h. The PVDF membranes were developed with enhanced chemiluminescent reagents (Beijing Applygen, P1050), and images were captured using a gel imager (Shanghai Tanon, 5200 Multi). β-Actin was used as an internal reference, and Image J software was utilized to analyze the relative protein expression levels. All uncropped images from western blots are shown in the Figs. S1–S3.

### Cell culture

Human SCs sNF96.2 (Wuhan Pricella, CL-0864) were seeded in a specialized medium for sNF96.2 (Wuhan Pricella, CM-0864) and incubated in a 37 °C cell incubator with an atmosphere of 95% O_2_ and 5% CO_2_. Cell identity was confirmed by S-100 immunofluorescence (IF) staining, and the absence of *mycoplasma* contamination was verified both by the manufacturer's certification and through monthly in-house testing during the study. SCs were passaged at a ratio of 1: 2, and those within 10 passages were selected for experiments.

### I-125 treatment and cell transfection

SCs were digested with 0.25% trypsin and seeded into a 12-well plate (3 × 10^4^ cells/well). Four I-125 seeds were placed in the four corners of a 6-well plate, and the 12-well plate was positioned directly above the 6-well plate containing the I-125 seeds. The plates were regularly rotated to ensure uniform radiation exposure to the cells. The cells were incubated for 44 h to receive a radiation dose of 2 Gy. 20 h after cellular exposure to I-125 radiation, 2 μg each of the pcDNA-NC, pcDNA-NFASC, pcDNA-CNTNAP2, and pcDNA-CTCF plasmids (Shanghai Genomeditech), along with Lipofectamine 3000 (Thermo Fisher Scientific, L3000015), were diluted separately with an equal volume of Opti-MEM. The DNA and 3 μl of Lipofectamine mixtures were combined, gently mixed, and incubated at room temperature for 15 min. The resulting mixture was then added dropwise to the cells under radiation conditions and co-incubated for 24 h. Cells were subsequently harvested to assess transfection efficiency.

### Measurement of lactate dehydrogenase (LDH) and malondialdehyde (MDA) levels

The levels of LDH and MDA in cells and facial nerve tissue were measured using LDH and MDA assay kits (Nanjing Jiancheng, A020-2-2, A003-1-2). Rabbit facial nerve tissue and SCs samples were collected separately. Following the manufacturer's instructions of the kits, LDH and MDA levels were determined in both the facial nerve tissue and cells.

### Detection of reactive oxygen species (ROS)

The 2′,7′-Dichlorodihydrofluorescein diacetate (DCFH-DA) fluorescent probe (Beijing Solarbio, 4091–99–0) was utilized to detect ROS in SCs and tissues. Facial nerve tissues were prepared into a single-cell suspension, suspended in serum-free medium, and adjusted to a cell count of 2 million cells per milliliter. The DCFH-DA fluorescent probe was added to achieve a final concentration of 5 μmol/L, followed by incubation at 37 °C for 30 min. The mixture was homogenized every 5 min to ensure adequate contact between the probe and the cells. Cells were rinsed to remove any DCFH-DA that had not entered the cells. Finally, the fluorescence intensity of ROS was determined using flow cytometry.

### Detection of mitochondrial membrane potential (MMP)

The MMP was determined using the JC-1 (Beijing Solarbio, M8650) staining method. SCs were suspended in fresh culture medium and seeded into a 6-well plate (1 × 10^5^ cells per well). After treatment with I-125 and transfection, 1 ml of JC-1 staining solution was added to each well and mixed evenly. The cells were incubated in a cell incubator for 20 min. Following incubation, the cells were washed with JC-1 staining buffer and observed under a fluorescence microscope (OLYMPUS).

### MitoSOX staining

The level of superoxide anion in cells was detected through the MitoSOX staining. The density of SCs was adjusted to 1 × 10^5^ cells/ml. MitoSOX Red working solution (MedChem Express, HY-D1055) was added to the cells, followed by incubation at room temperature in the dark for 30 min, with mixing every 5 min to ensure sufficient contact between the cells and the probe. The cells were washed with culture medium to remove any residual MitoSOX dyestuff outside the cells. After staining the cell nuclei with DAPI for 5 min, the cells were observed under a fluorescence microscope.

### Assessment of cell proliferation using cell counting kit-8 (CCK-8)

SCs were seeded into a 96-well plate at a density of 5 × 10^3^ cells per well. After treatment with I-125 seeds and transfection, 10 μl of CCK-8 reagent (TargetMol, C0005) was added to each well. The cells were incubated in the dark for 2 h. The optical density at 450 nm was measured using a microplate reader.

### Transwell assay

SCs cells were digested with 0.25% trypsin (Gibco, 25200072), resuspended in serum-free media, and adjusted to a cell density of 1 × 10^5^ cells/ml. The 100 μl of cell suspension was added to each well of the Transwell chamber, while the lower chamber was filled with media containing 20% fetal bovine serum (ExCell, FSP500). The Transwell chambers were incubated in a cell culture incubator for 48 h. Cells in the lower chamber were fixed with 4% paraformaldehyde for 30 min, stained with crystal violet for 15 min, washed with phosphate buffer saline (PBS, Beijing ZHGB-Bio, ZLI-9062), and finally observed and photographed under a microscope. For each replicate, three fields of view were randomly selected, numbered, and then shuffled randomly. A staff member not involved in the experiment performed the cell counting, counting only those cells whose nuclei were entirely located in the lower chamber.

### Co-Immunoprecipitation (Co-IP) assay

SCs were washed with PBS, and lysed using IP lysis buffer containing protease inhibitors and phosphatase inhibitors. The supernatant was collected after centrifugation. Input, IgG, and NFASC groups were set up. A small amount of cell lysate was taken as the Input group. For the IgG and NFASC groups, 1 μg of IgG and NFASC (Biomatik, CAU21375) antibodies were added to the respective cell lysates, and the mixtures were incubated overnight at 4 °C. Then, 25 μl of Protein A/G magnetic beads (Thermo Fisher Scientific, 88,804, Waltham, MA, USA) were washed with washing buffer, blocked with 5% skim milk for 30 min, washed again, and resuspended in binding buffer. The beads were then incubated overnight with the cell lysates from the Input, IgG, and NFASC groups, respectively. The next day, the lysis products were magnetically separated from the beads, and the beads were rinsed. Loading buffer was added to the beads, followed by a 10-min boiling water bath. After magnetic separation, the supernatant was collected as the protein sample. Western blotting was performed to detect CNTNAP2 bound to NFASC.

### IF staining of SCs

After trypsin digestion and PBS washing, SCs were seeded into 6-well plates. Coverslips were placed into the cell culture wells and incubated for 24 h. The cells on the coverslips were fixed with 4% paraformaldehyde for 20 min, followed by permeabilization with 0.5% Triton-X100 for 10 min. The cells were blocked with 3% bovine serum albumin for 1 h before incubation with primary (Rabbit anti-CNTNAP2 Shanghai Abcam, ab153856; mouse anti-NFASC NeuroMab, ABI-75–027) and secondary antibodies. After washing, the coverslips were mounted with a fluorescent mounting medium containing DAPI. Images were captured and recorded under a fluorescence microscope.

### Dual-luciferase reporter assay

SCs were seeded in a 24-well plate at a density of 2 × 10^4^ cells per well and transfected with pcDNA-NC and pcDNA-CTCF for 24 h. Cells were collected, and the activities of Renilla luciferase and firefly luciferase were measured using a dual-luciferase reporter assay kit (Shanghai Beyotime, RG027) according to the manufacturer's instructions. The activity of Renilla luciferase served as an internal control.

### CUT&RUN for PCR

SCs were digested, centrifuged, washed with washing buffer, and resuspended. The cells were incubated with 10 μl of ConA beads (Nanjing Vazyme, HD102–01) for 10 min, followed by centrifugation and discarding of the supernatant. The cell-bead complexes were incubated with primary antibodies (abcam, ab128873) overnight at 4 °C. A 1 μl aliquot of pG-MNase enzyme (Nanjing Vazyme, HD102–01) was diluted in 100 μl of MNase Dilution Buffer, and 1 μl of the resulting mixture was added to 100 μl of Dig-washing buffer and mixed thoroughly for subsequent use. Following incubation with the primary antibodies, the reaction mixture was centrifuged and the supernatant was discarded. The pellet was washed multiple times with Dig-washing buffer. Subsequently, 100 μl of the pG-MNase premix was added to the reaction mixture, mixed well, and incubated for 1 h. After rinsing the mixture with Dig-washing buffer, a CaCl_2_ premix was added for incubation, and the supernatant was discarded. The stop buffer was used to terminate fragmentation, and the supernatant obtained after centrifugation represented the chromatin, which was purified using a purification column. The binding of CTCF to the CNTNAP2 promoter was detected by real-time reverse transcriptase-polymerase chain reaction (RT-qPCR).

### RT-qPCR

SCs were lysed using TRIzol to extract total RNA from the cells, and the RNA concentration was determined using an ultra-micro spectrophotometer. The first strand of cDNA was synthesized using a FastKing RT Kit FastKing cDNA First-Strand Synthesis Kit (Beijing Tiangen, KR116–02). RT-qPCR reactions were performed using the Taq Pro Universal SYBR qPCR Master Mix (Nanjing Vazyme, Q712–02). The amplification reaction was performed in a 10 μl system containing 5 μl of Taq Pro Universal SYBR qPCR Master Mix, 0.25 μl of forward primer, 0.25 μl of reverse primer, and 1 μl of cDNA template, with the final volume adjusted to 10 μl using RNase-free ddH_2_O. The PCR cycling conditions were set as follows: initial denaturation at 95 °C for 10 min, denaturation at 95 °C for 10 s, annealing and extension at 60 °C for 30 s, for a total of 40 cycles. Data were compiled, and the relative expression levels of the target genes were analyzed using the 2^-ΔΔCt^ method. The primer sequences were listed in Table 1.

**Table 1:** Primer sequences

Gene	Primer sequences (F: forward primer, R: Reversed primer)
β-actin	F:GCAGGAGTACGATGAGTCCG
	R:ACGCAGCTCAGTAACAGTCC
CTCF	F:CAGCATTCCTATATTGAG
	R:GTCTACAAGCGTAATCAC
CNTNAP2	F:AATTTGGGCAATGTGGAG
	R:CTCATCTTGGTCTGTGTG
NFASC	F:CATCCTGATTGAATGTGAAG
	R:TTGAAGAACCTGCTGTTG

### Transmission electron microscope (TEM)

The rabbit facial nerve was fixed in 2.5% glutaraldehyde fixative solution (Wuhan Servicebio, G1102–100 Ml), followed by fixation with 1% osmium tetroxide at room temperature for 2 h and subsequent washing with PBS. The tissue was dehydrated with ethanol and embedded in a mixture of acetone and embedding medium overnight. On the following day, the tissue was transferred to a dry centrifuge tube, embedded in epoxy resin, and polymerized at 70 °C overnight. After slicing with a microtome, the sections were stained with 3% uranyl acetate and lead citrate for 8 min, and the excess stain was washed off. The water was absorbed with filter paper, and the staining results were observed under a TEM.

### Tissue embedding, sectioning, and Luxol fast blue (LFB) staining

The rabbit facial nerve was fixed with 4% paraformaldehyde, dehydrated, paraffin-embedded, and sectioned. The sections were dewaxed and hydrated with ethanol, followed by washing with distilled water. They were stained with fast blue staining solution (Beijing Solarbio, G3245) at room temperature overnight. Excess staining solution was washed off with 95% ethanol, and the sections were rinsed with distilled water. The sections were differentiated with lithium carbonate for 3 to 5 s, rinsed with distilled water again, rapidly dehydrated with ethanol, cleared with xylene, and mounted with neutral balsam. Finally, the sections were observed and photographed under a microscope.

### IF staining of the facial nerve

The IF experiment was conducted to detect the fluorescence intensity of S100β, NeuN, and Ki67 in the facial nerve. Paraffin sections were dewaxed with xylene and ethanol, rinsed with PBS solution, and treated with citrate buffer (Beijing ZHGB-Bio, ZLI-9064) for antigen retrieval. The sections were incubated with 5% goat serum at 37 °C for 1 h, followed by incubation with primary antibodies (S100β 1: 100, Beijing Bioss bs-1248R; Neun 1: 100, Shanghai Abcam ab177487; Ki67 1: 100, Beijing Bioss, bsm-10837M) diluted in 2% goat serum at 4 °C overnight in the dark, and secondary antibodies (488 R/CY3M 1: 800) at 37 °C for 60 min in the dark. After rinsing, the sections were mounted with a fluorescent mounting medium containing DAPI and observed under a fluorescent microscope.

### Statistical analysis

Each group in the aforementioned experiments was repeated at least three times. The data are expressed as "mean ± standard deviation." Data analysis, statistical processing, and graph were performed using GraphPad Prism software. Normality of the data was assessed using the Shapiro-Wilk test. For comparisons among multiple groups, one-way ANOVA was performed, and the least significant difference test was used for *post hoc* pairwise comparisons. A q-value < 0.05 was considered statistically significant.

## Ethics

The animal experiment in this study was approved by the Experimental Animal Ethics Committee of Kunming Medical University (kmmu20230921).

## Data availability

The RNA-seq data generated in this study is available at the NCBI GEO depository under the accession number GSE312643 (https://www.ncbi.nlm.nih.gov/geo/query/acc.cgi?acc=GSE312643).

## Supporting information

This article contains supporting information.

## Conflict of interest

The authors declare that they have no conflicts of interest with the contents of this article.
