# Supplementary material for: Neurofascin stabilization by contactin-associated protein-like 2 and CTCF alleviates mitochondrial dysfunction in Schwann cells during facial nerve injury
Source: J Biol Chem. 2025 Dec 22;302(2):111090. doi: 10.1016/j.jbc.2025.111090 (PMC12818255; doi:10.1016/j.jbc.2025.111090)
Supplement: Supporting information [file mmc1.docx]

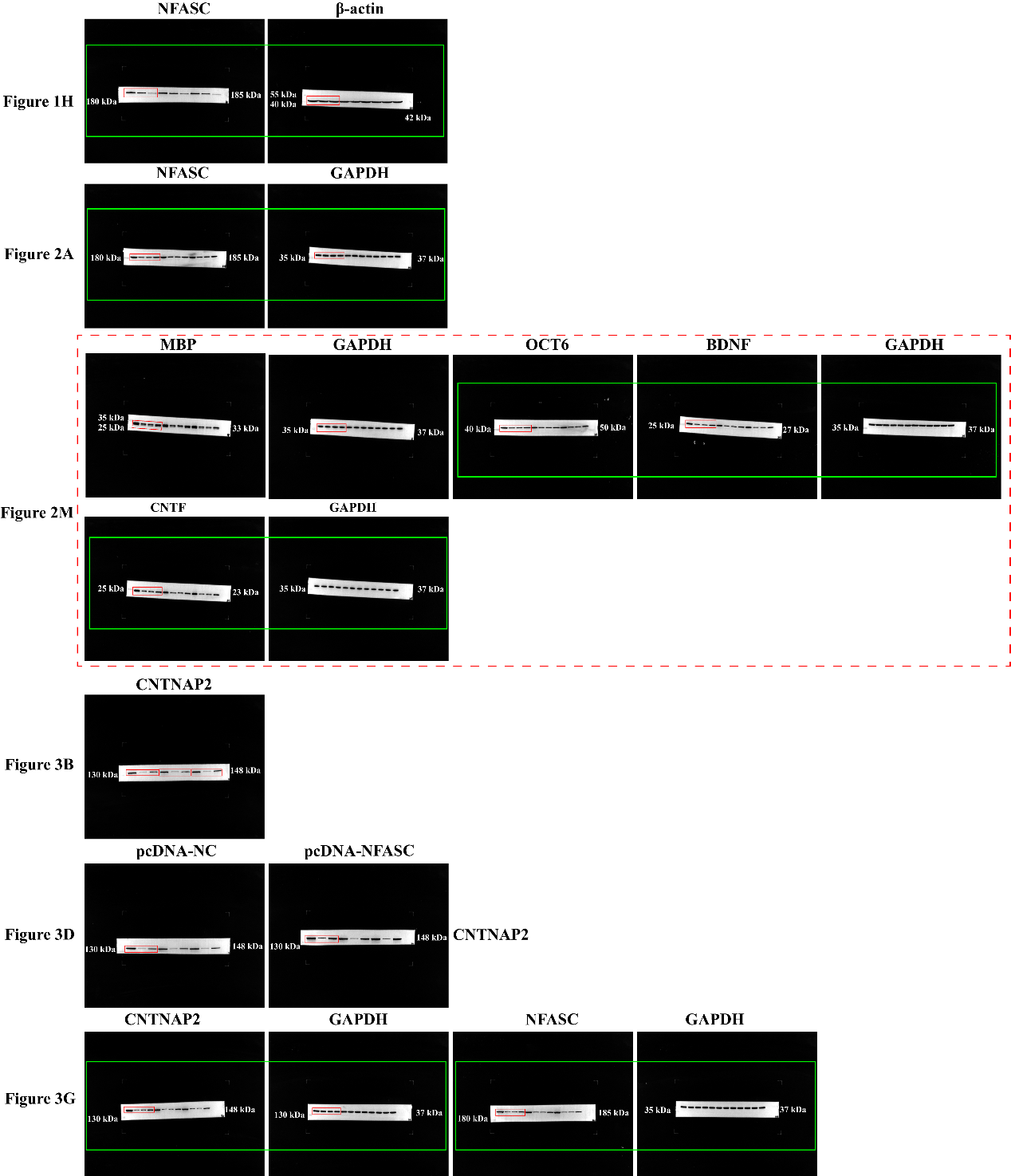


Supplementary figure 1 Full and uncropped western blots for Figure 1- Figure 3


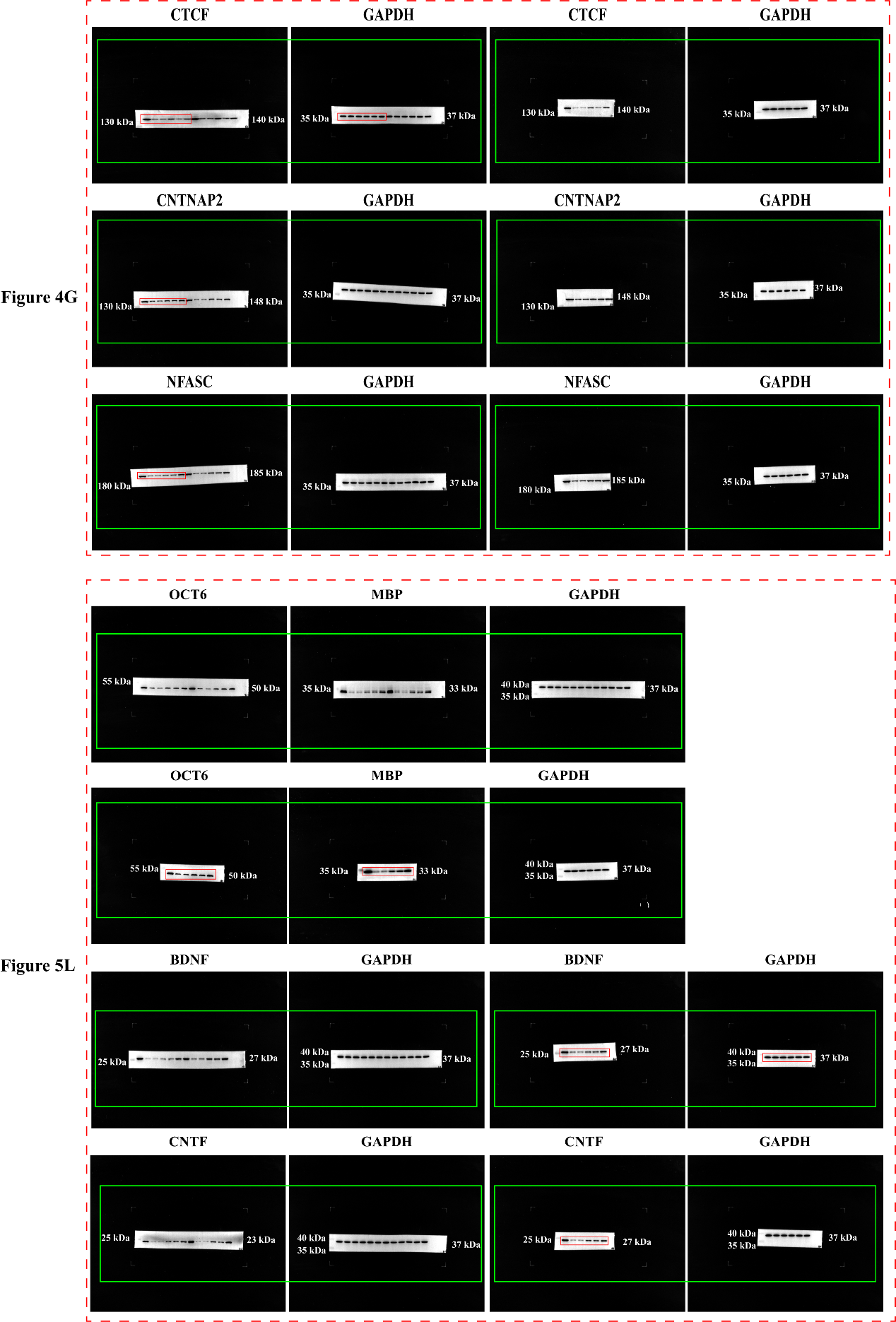


Supplementary figure 2 Full and uncropped western blots for Figure 4- Figure 5


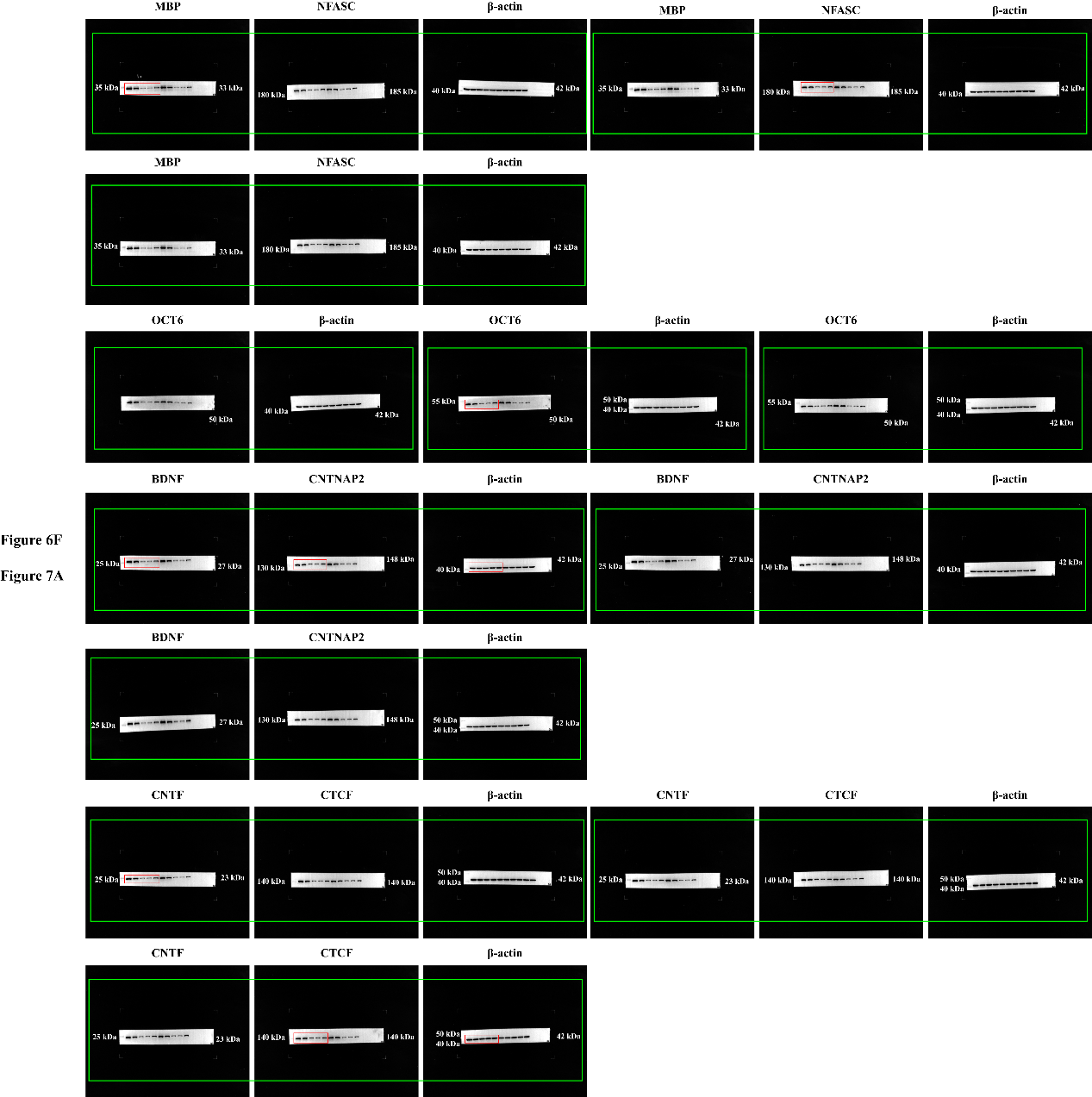


Supplementary figure 3 Full and uncropped western blots for Figure 6- Figure 7
